# Dietary *Laminaria japonica* Polysaccharide Alleviates Aged-Maize-Associated Intestinal Oxidative Stress and Systemic Inflammation in Hu Sheep: Associations with Cecal Microbiome–Metabolome Remodeling

**DOI:** 10.3390/ani16142146

**Published:** 2026-07-10

**Authors:** Jiaxuan Dong, Shuhan Li, Jiamei Song, Yuansheng Ma, Hangshu Xin, Yonggen Zhang, Guangning Zhang

**Affiliations:** College of Animal Science and Technology, Northeast Agricultural University, Harbin 150030, China; s240502140@neau.edu.cn (J.D.); s2505012014@neau.edu.cn (S.L.); songjiamei1120123@163.com (J.S.); yuanshengma@126.com (Y.M.); hangshu.xin@neau.edu.cn (H.X.); zhangyonggen@neau.edu.cn (Y.Z.)

**Keywords:** aged maize, *Laminaria japonica* polysaccharide, Hu sheep, intestinal oxidative stress, systemic inflammation, hepatic antioxidant status, cecal microbiome–metabolome remodeling

## Abstract

Long-term maize storage causes oxidative deterioration, but its effects on intestinal redox status, systemic inflammation, and liver-related responses in ruminants remain unclear. *Laminaria japonica* polysaccharide (LJP) has antioxidant, immunomodulatory, and microbiota-regulating properties, but its efficacy during aged-maize feeding is unknown. This study evaluated whether LJP mitigates oxidative and inflammatory responses in Hu sheep fed aged maize and characterized cecal microbiome and metabolome alterations. Twenty-one Hu sheep (39.05 ± 3.55 kg) were assigned to three diets (*n* = 7) and fed for 10 weeks (a 14-day adaptation period followed by 8 weeks of treatment): normal maize (CK), aged maize (AM), or aged maize with 0.5% LJP (AML). Compared with CK, AM increased plasma lipopolysaccharide (0.428 vs. 0.379 EU/mL), TNF-α, and IL-1β, and raised ileal reactive oxygen species (248.79 vs. 166.23 fluorescence intensity/mg; *p* < 0.001) and malondialdehyde (1.87 vs. 1.63 nmol/L; *p* = 0.006), consistent with systemic inflammation and intestinal oxidative stress. AML lowered these inflammatory and oxidative indices and increased hepatic T-AOC (*p* = 0.009) and catalase activity (*p* = 0.013). Integrated 16S rRNA and untargeted metabolomic analysis revealed treatment-associated cecal microbe–metabolite associations. These findings indicate that aged-maize feeding was associated with intestinal and systemic redox–inflammatory changes in Hu sheep, whereas dietary LJP was associated with partial mitigation, potentially involving microbial and metabolic remodeling.

## Simple Summary

Maize is one of the most important grains used to feed farm animals, but grain that has been stored for several years can slowly spoil as its fats break down, even when it still looks normal. We wanted to know whether feeding with this older maize harms the health of sheep, and whether LJP (*Laminaria japonica* polysaccharide) taken from edible brown seaweed could help protect them. We fed young male sheep one of three diets: normal maize, several-year-old maize, or aged maize with a small amount of seaweed extract added. We then examined their blood, liver, and gut and the natural community of microbes living in their intestine. Sheep eating the aged maize showed more signs of inflammation throughout the body and more damage in the gut, suggesting the spoiled grain stressed their digestive and overall health. Adding the seaweed extract reduced this inflammation and damage and strengthened the animals’ natural defenses against it. It also shifted the balance of gut microbes and the chemicals they produce. These findings suggest that a simple seaweed supplement may help farmers safely use stored maize while keeping their animals healthier.

## 1. Introduction

Maize remains a cornerstone energy ingredient in ruminant production because its high starch density, palatability, and storability make it central to year-round ration formulation. However, prolonged storage can reduce the nutritional predictability of maize. Aged maize may appear visually intact but can undergo biochemical deterioration, including changes in oxidation-related indices, fat acidity, starch and protein retention, and overall physicochemical quality across storage duration and environment [1,2]. Therefore, aged maize should not be regarded simply as old grain, but as a feed ingredient whose biological effects may differ from those of well-preserved maize.

Among the potential biological concerns associated with aged maize, oxidative deterioration is particularly relevant. Because maize contains oxidation-prone lipid fractions, prolonged storage can favor lipid hydrolysis and oxidation-related product accumulation. These changes are not only indicators of quality loss but may also influence redox and inflammatory responses after ingestion. In monogastric models, naturally oxidized maize oil has been reported to impair intestinal health, reshape cecal microbial structure, and alter hepatic lipid metabolism, suggesting that oxidized maize-derived lipid fractions can act as gut and liver stressors rather than simply inferior energy sources [3,4,5]. In addition, long-term storage may increase the risk of storage-associated contaminants, including mycotoxins, although this risk depends on storage conditions, grain handling, and actual contaminant levels [6,7,8]. Therefore, safety-related characterization of aged maize is necessary when evaluating its physiological effects in animals. Although direct evidence in ruminants remains comparatively limited, feeding oxidatively damaged or spoiled feedstuffs to sheep and cattle has likewise been associated with oxidative stress, inflammatory activation, and hindgut microbial disturbance, indicating that the physiological consequences of dietary oxidation are not restricted to monogastric species [9,10].

In ruminants, the hindgut is highly responsive to diet-driven perturbations. Previous studies in sheep have shown that nutritional challenges can be accompanied by cecal or colonic microbial remodeling, increased lipopolysaccharide (LPS) exposure, inflammatory activation, and mucosal injury [9,10]. Dietary oxidative stressors and microbial disturbances may disrupt intestinal redox balance and increase systemic exposure to microbial products such as LPS. Because gut-derived microbial products and metabolites reach the liver through the portal circulation, intestinal disturbances can also be reflected in hepatic oxidative, inflammatory, or hepatobiliary-related responses [11,12,13,14]. However, whether aged maize with storage-related quality deterioration affects intestinal redox status, systemic inflammatory tone, and liver-related biochemical responses in sheep remains unclear.

These considerations make microbiota-targeted antioxidant interventions attractive under aged-maize feeding conditions. Marine macroalgal polysaccharides have attracted attention as functional feed additives because they possess antioxidant and immunomodulatory properties and may interact with the gut microbiota. Among these candidates, *Laminaria japonica* polysaccharide (LJP) is of particular interest. Emerging evidence indicates that LJP and related fractions can alleviate metabolic liver injury through gut microbiota regulation, improve systemic antioxidant and health-related phenotypes through coordinated microbiome–metabolome remodeling, and behave as a prebiotic-like substrate that persists through the gastrointestinal tract and increases short-chain fatty acid production [15,16,17,18,19,20]. However, whether dietary LJP can alleviate aged-maize-associated physiological disturbances in ruminants has not been determined. In ruminants, marine macroalgal polysaccharides have been investigated mainly in the context of rumen fermentation and enteric methane mitigation, whereas their effects on intestinal redox status, systemic inflammation, and hindgut microbial ecology remain largely unexplored.

Against this background, we hypothesized that replacing normal maize with aged maize would be associated with intestinal oxidative stress, systemic inflammatory responses, and liver-related biochemical changes in Hu sheep, whereas dietary LJP supplementation would partially mitigate these responses. To our knowledge, no study has yet characterized the intestinal redox, systemic inflammatory, and liver-related biochemical responses to aged-maize feeding in a ruminant, or tested whether a marine algal polysaccharide can mitigate such responses. The present study addresses this gap by combining systemic, intestinal, and hepatic redox–inflammatory phenotyping with cecal microbiome–metabolome profiling in Hu sheep. Therefore, this study aimed to evaluate the effects of aged-maize feeding and LJP supplementation on plasma inflammatory biomarkers, intestinal and hepatic antioxidant status, and plasma biochemical traits, and to explore whether these responses were accompanied by changes in cecal microbial and metabolic profiles using 16S rRNA gene sequencing, untargeted metabolomics, and microbiome–metabolome association analysis.

## 2. Materials and Methods

### 2.1. Feed Ingredients and Laminaria japonica Polysaccharide

Normal maize and aged maize were purchased from grain depots in the Harbin area (Heilongjiang, China). Normal maize had been stored for approximately 1 year, whereas aged maize had been stored for approximately 4 years under conditions consistent with the national seed/grain storage guideline (GB/T 7415-2008) [21], including dry, ventilated storage and standardized stacking practices. The moisture content of all maize lots was <13%, and the contents of mycotoxins, heavy metals, and other regulated contaminants were below the maximum limits specified in the Hygienical Standard for Feeds (GB 13078-2017) [22] (Table 1).

*Laminaria japonica* polysaccharide (LJP; purity 98%, sulfate group content 28.9%) was supplied as a dry powder by Qingdao Mingyue Seaweed Bio-Health Technology Group (Qingdao, China). Brown-seaweed sulfated polysaccharides have attracted attention as functional feed additives due to their antioxidant and immunomodulatory properties and their potential prebiotic effects on the gut microbiota.

### 2.2. Safety-Related Characterization of Aged Maize

Compared with normal maize, aged maize showed a markedly higher fatty acid value (84.15 vs. 53.30 mg KOH/100 g), indicating storage-related lipid hydrolysis and oxidative deterioration. To exclude acute contaminant toxicity as a confounder, a comprehensive safety panel—covering mycotoxins, heavy metals and other inorganic contaminants, organochlorine and pesticide residues, antinutritional factors, and microbiological indicators—was determined by a qualified third-party laboratory using national standard methods. All indicators were within the limits of the Hygienical Standard for Feeds (GB 13078-2017) (Table 1), indicating that the aged maize was oxidatively deteriorated rather than contaminated above regulatory limits.

### 2.3. Animals, Ethics Statement, and Experimental Design

The Animal Ethics Committee of Northeast Agricultural University approved all animal procedures in this study, which were conducted in accordance with institutional guidelines for the care and use of laboratory animals. The feeding trial was conducted from June to August 2023 at the Acheng Experimental Base of Northeast Agricultural University, Harbin, China.

Twenty-one healthy Hu sheep with similar initial body weights were enrolled in the study (age, 161 ± 4.3 d; initial body weight, 39.05 ± 3.55 kg). Spanning 10 weeks in total, the trial comprised a 14-day adaptation phase followed by a formal feeding period of 8 weeks. During the adaptation period, the concentrate-to-forage ratio was gradually adjusted from 0:10 to 7:3. Following the adaptation period, the sheep were allocated to three dietary treatments according to their initial body weight, resulting in seven sheep in each treatment group: CK, a control diet containing normal maize; AM, a diet in which normal maize was completely replaced by aged maize; and AML, the AM diet supplemented with 0.5% *Laminaria japonica* polysaccharide (LJP) on a dry matter (DM) basis. The 0.5% LJP dose was selected according to previous research and preliminary animal observations. The individual lamb was considered the experimental unit for all biochemical, inflammatory, microbiome, and metabolomic analyses.

### 2.4. Diet Formulation and Feeding Management

Every diet was formulated on a dry matter basis. The experimental diets were formulated to meet the nutrient requirements of growing Hu sheep according to the National Research Council recommendations [23]. Table 2 presents the ingredient composition together with the analyzed nutrient levels (DM basis). The forage source was Chinese wildrye hay, and the concentrate portion included maize (normal or aged), sprayed maize bran, maize germ meal, DDGS, soybean meal, molasses, and a mineral–vitamin premix.

Before the trial, the barn was thoroughly disinfected and sheep were dewormed. Animals were housed individually in pens (1 m × 2 m) bedded with litter, which was replaced regularly to maintain hygiene. Sheep were fed twice daily at 07:00 and 17:00. Forage was offered first, and concentrate was provided after forage intake was completed. Fresh water was available ad libitum throughout the trial. Feed was offered to allow 5–10% refusals to ensure ad libitum intake conditions.

### 2.5. Sample Collection and Processing

When the feeding trial was completed, the sheep were slaughtered humanely following institutional guidelines. On the slaughter day, jugular vein blood was drawn into heparinized vacuum tubes and subjected to centrifugation at 3000× *g* to separate the plasma, which was then divided into aliquots and kept at −20 °C pending the measurement of plasma biochemical and redox-related indicators. Liver tissue (about 2–3 g) was obtained immediately following evisceration, washed in ice-cold saline to eliminate residual blood, blotted dry, frozen rapidly in liquid nitrogen, and maintained at −80 °C for the assessment of antioxidant status. Likewise, representative portions of the ileum and colon were excised, lightly flushed with ice-cold saline to clear luminal contents, blotted dry, snap-frozen in liquid nitrogen, and held at −80 °C for evaluating oxidative stress and antioxidant markers, namely superoxide dismutase (SOD), glutathione peroxidase (GSH-Px), total antioxidant capacity (T-AOC), malondialdehyde (MDA), and reactive oxygen species (ROS). Furthermore, cecal digesta were collected under aseptic conditions into sterile cryovials, snap-frozen in liquid nitrogen without delay, and preserved at −80 °C for later 16S rRNA gene sequencing and untargeted metabolomic profiling.

### 2.6. Plasma Biochemical Profiling

Plasma samples were sent to Huaying Biotechnology Institute (Beijing, China), where total cholesterol (TC), triglycerides (TG), high-density lipoprotein cholesterol (HDL), low-density lipoprotein cholesterol (LDL), aspartate aminotransferase (AST), alanine aminotransferase (ALT), total bilirubin (TBIL), direct bilirubin (DBIL), and alkaline phosphatase (ALP) were measured using an automatic biochemical analyzer (Beckman Coulter AU680, Brea, CA, USA). Commercial assay kits supplied by Biosino Bio-Technology and Science Inc. (Beijing, China) were used for the biochemical assays, with the following catalogue numbers: TC, 100020080; TG, 100020090; HDL, 100020235; LDL, 100020245; AST, 100020010; ALT, 100020000; TBIL, 100020125; DBIL, 100020130; and ALP, 100020020.

### 2.7. Systemic Inflammatory Biomarkers

Plasma inflammatory biomarkers, including lipopolysaccharide (LPS), tumor necrosis factor-α (TNF-α), and interleukin-1β (IL-1β), were determined using commercially available assay kits supplied by Huaying Biotechnology Institute (Beijing, China; LPS, cat. no. HY-H0042; TNF-α, cat. no. HY-H0019; IL-1β, cat. no. HY-H0001), in accordance with the manufacturer’s instructions. All plasma samples were analyzed by Huaying Biotechnology Institute as a third-party testing service, following standardized procedures for sheep cytokine and endotoxin quantification.

### 2.8. Antioxidant Capacity and Oxidative Stress Indices in Liver and Intestinal Tissues

For liver, ileum, and colon tissues, antioxidant capacity and oxidative stress indices were determined using commercially available assay kits supplied by Huaying Biotechnology Institute (Beijing, China), following the manufacturer’s protocols. The catalogue numbers of the kits were as follows: superoxide dismutase (SOD), HY-M0001; malondialdehyde (MDA), HY-M0003; glutathione peroxidase (GSH-Px), HY-M0004; reactive oxygen species (ROS), HY-M0087; total antioxidant capacity (T-AOC), HY-M0011; and catalase (CAT), HY-M0018.

Frozen liver and intestinal tissues were processed under ice-cold conditions. Briefly, tissues were homogenized in pre-chilled saline or assay buffer, and the supernatant obtained after centrifugation was used for biochemical assays. Protein concentration was determined where required by the kit instructions to normalize fluorescence- or enzyme-based readouts. T-AOC was measured by reading the absorbance at 520 nm; GSH-Px activity was measured at 412 nm; SOD activity was measured at 450 nm; CAT activity was measured at 405 nm using the ammonium molybdate method; MDA concentration was determined using the thiobarbituric acid reactive substance method at 532 nm; and ROS levels in the ileum and colon were determined using a fluorescent probe-based method and expressed as fluorescence intensity per unit tissue or protein.

### 2.9. 16S rRNA Gene Amplicon Sequencing and Bioinformatic Analysis of Cecal Digesta

Total genomic DNA was isolated from cecal digesta with the MagBeads FastDNA Kit for Soil (MP Biomedicals, Irvine, CA, USA), following the protocol provided by the manufacturer. The concentration and purity of the extracted DNA were determined on a NanoDrop NC2000 spectrophotometer, while its integrity was checked through 0.8% agarose gel electrophoresis.

Amplification of the V3–V4 hypervariable regions within the bacterial 16S rRNA gene was carried out using the primers 338F (5′-barcode+ACTCCTACGGGAGGCAGCA-3′) and 806R (5′-GGACTACHVGGGTWTCTAAT-3′), in which sample-specific barcodes were attached to the forward primer. Each PCR reaction had a final volume of 25 μL, comprising 0.25 μL Q5 high-fidelity DNA polymerase, 5 μL 5× reaction buffer, 5 μL 5× high GC buffer, 2 μL dNTPs (10 mM), 2 μL template DNA, 1 μL of each forward and reverse primer (10 μM), and 8.75 μL nuclease-free water [24]. Thermal cycling proceeded as follows: an initial denaturation step at 98 °C for 5 min, then 25 cycles consisting of 98 °C for 30 s, 52 °C for 30 s, and 72 °C for 45 s, and a final extension at 72 °C for 5 min before holding at 12 °C.

Amplification products were confirmed on 2% agarose gels, after which they were purified and quantified with the Quant-iT PicoGreen dsDNA Assay Kit (Thermo Fisher Scientific, Waltham, MA, USA). The purified amplicons were combined in equimolar quantities, and libraries were prepared using the Illumina TruSeq Nano DNA LT Library Prep Kit (Illumina, San Diego, CA, USA). Sequencing of the resulting libraries was performed on an Illumina NovaSeq platform under a paired-end 250 bp (PE250) scheme (Illumina, San Diego, CA, USA).

Raw sequencing data were processed using QIIME2 (version 2024.5) [25]. Demultiplexed reads were trimmed to remove primer sequences using the cutadapt plugin and then subjected to quality filtering, denoising, paired-end merging, and chimera removal using DADA2 [26], generating amplicon sequence variants (ASVs) and the corresponding abundance table. Representative ASV sequences were aligned using MAFFT 7.520 [27], and a phylogenetic tree was constructed using FastTree2 [28]. Taxonomic assignment was performed using the classify-sklearn naïve Bayes classifier against the Greengenes reference database [29,30,31].

Based on the ASV table, alpha-diversity indices were computed, comprising Chao1, observed species, Shannon, Simpson, Faith’s phylogenetic diversity, Pielou’s evenness, and Good’s coverage. To illustrate how the cecal microbial communities were distributed overall across treatments, beta-diversity patterns were displayed through principal coordinate analysis (PCoA) built on Bray–Curtis distance. Differences in overall community structure among treatments were tested by permutational multivariate analysis of variance (PERMANOVA, adonis) on Bray–Curtis distances with 999 permutations, followed by pairwise PERMANOVA with Benjamini–Hochberg (BH) false-discovery-rate correction. Homogeneity of multivariate dispersion among groups was assessed using PERMDISP. Relative abundance plots were employed to present the taxonomic composition at various levels. Microbial taxa that differed among treatments were detected by linear discriminant analysis effect size (LEfSe), where an LDA score above 2.0 served as the cutoff for discriminative taxa. Since LEfSe functions as a biomarker discovery method, the taxa it identified were regarded as microbial markers associated with the treatments rather than confirmed causal drivers.

Microbial functional profiles were predicted using PICRUSt2 based on ASV representative sequences [32]. Predicted KEGG Orthologs and pathways were normalized by 16S rRNA gene copy number. Differential predicted pathways among treatments were identified using the Kruskal–Wallis test followed by Benjamini–Hochberg FDR correction, and pathways with adjusted q < 0.05 were considered significantly different.

### 2.10. Untargeted Metabolomic Analysis of Cecal Digesta

Untargeted metabolomic profiling of the cecal digesta was conducted on an ultra-high-performance liquid chromatography–mass spectrometry (UHPLC-MS) platform. In brief, a 20 mg portion of each cecal digesta sample was placed into a 2 mL centrifuge tube, to which 300 μL of precooled methanol containing 5 ppm L-2-chlorophenylalanine was introduced, serving simultaneously as the extraction solvent and the internal standard. After two steel beads were added, the mixture underwent 30 s of vortexing and was then homogenized at 55 Hz for 60 s in a high-throughput tissue grinder. To achieve adequate metabolite recovery, this homogenization was carried out a second time. The samples were next sonicated for 10 min, held at −20 °C for 30 min, and centrifuged at 12,000 rpm for 10 min at 4 °C. The resulting supernatants were retrieved and passed through a 0.22 μm membrane filter prior to LC-MS analysis. Quality control (QC) samples were prepared by combining aliquots from all sample extracts, allowing the analytical stability and reproducibility to be monitored.

The metabolomic analysis relied on a Thermo Vanquish Flex UHPLC system connected to a Thermo Orbitrap Exploris 120 high-resolution mass spectrometer. Separation was accomplished on an ACQUITY UPLC HSS T3 column (Waters, Milford, MA, USA, 100 Å, 1.8 μm, 2.1 mm × 100 mm). Operating parameters were a flow rate of 0.4 mL/min, a column temperature of 40 °C, an autosampler temperature of 8 °C, and an injection volume of 2 μL. Phase A of the mobile phase was 0.1% formic acid in water, whereas phase B was acetonitrile with 0.1% formic acid. Gradient elution followed this program: 5% B from 0 to 1.0 min; a linear rise to 95% B between 1.0 and 4.7 min; 95% B from 4.7 to 6.0 min; a drop to 5% B during 6.0–6.1 min; and 5% B from 6.1 to 8.5 min.

Mass spectra were collected in both positive and negative ion modes through a data-dependent acquisition approach. Values for the sheath gas, auxiliary gas, capillary temperature, and auxiliary gas heater temperature were 40 arb, 10 arb, 320 °C, and 300 °C, respectively. Full-scan MS data were obtained at a resolution of 60,000 across an m/z range of 70–1000, with the maximum injection time set to 100 ms. The four most abundant precursor ions were chosen for MS/MS fragmentation, applying a dynamic exclusion window of 4 s. MS/MS spectra were recorded at a resolution of 15,000, employing a higher-energy collisional dissociation energy of 30% together with an automatic maximum injection time. Before the formal analysis began, 2–4 QC injections were run to equilibrate the LC-MS system, and one QC sample was inserted after every 5–10 study samples over the course of the analytical run [33].

Raw MS data were processed using MS-DIAL software version 4.9.221218 for peak detection, deconvolution, alignment, gap filling, normalization, and metabolite annotation. Low-quality features were removed according to QC- and blank-based filtering criteria. Specifically, features with a relative standard deviation greater than 30% in QC samples were excluded, and features undetected in more than 50% of QC samples or detected in less than 50% of biological samples within any treatment group were removed. Features with substantially higher signals in blank samples than in biological samples were considered background contaminants and were excluded from downstream analysis. Remaining missing values were imputed using the gap-filling function implemented in MS-DIAL or half of the minimum positive value. Peak intensities were normalized to the internal standard and total ion current, followed by log transformation and Pareto scaling before multivariate and univariate statistical analyses. The stability of the internal standard and the clustering pattern of QC samples in principal component analysis were examined to evaluate analytical reproducibility. When necessary, QC-based signal correction was applied to minimize batch effects and analytical drift during LC-MS acquisition.

Metabolite annotation was carried out against the PSNGM database, with additional support from the mzCloud, LIPID MAPS, HMDB, MoNA, and NIST 2020 MS/MS libraries as well as AI-predicted MS/MS spectral libraries. The principal annotation parameters were configured as follows: MS1 identification tolerance = 0.01 Da, MS2 identification tolerance = 0.05 Da, smoothing level = 3, minimum peak height = 10,000, minimum peak width = 5, mass slice width = 0.05 Da, and identification score cut-off = 70. Metabolite annotations that were not verified by authentic standards were treated as putative identifications, resting on accurate mass, MS/MS spectral matching, and database annotation.

### 2.11. Integrated Microbiome–Metabolome Association Analysis

To investigate the associations between cecal microbial alterations and metabolic changes, an integrated microbiome–metabolome analysis was performed using correlation-based methods. Microbial relative abundance data at the genus level and differential metabolite features identified from cecal digesta were included in the analysis. Spearman’s correlation coefficients were calculated between differential genera and differential metabolites, and associations were considered significant at |r| ≥ 0.60 and *p* < 0.05. For graphical presentation, the top 20 differential genera and top 20 differential metabolites, ranked by the magnitude of intergroup differences, were selected for visualization. Circos chord diagrams were constructed to display the direction and strength of significant microbiota–metabolite correlations and to annotate the upregulated or downregulated metabolite features in each comparison. This integrative analysis was used to identify potential microbe-associated metabolic shifts in the cecum in response to dietary treatments.

### 2.12. Data Processing and Statistical Analysis

All raw data were initially checked and organized using Microsoft Excel 2021 and then analyzed using SAS software version 9.2 (SAS Institute Inc., Cary, NC, USA). The individual lamb was considered the experimental unit for all biochemical, inflammatory, antioxidant, microbiome, and metabolomic analyses. Data for plasma biochemical parameters, inflammatory biomarkers, and antioxidant indices in liver and intestinal tissues are presented as means with the standard error of the mean (SEM), unless otherwise stated.

For the plasma biochemical parameters, inflammatory biomarkers, and antioxidant indices measured in liver and intestinal tissues, the normality of residuals was checked with the Shapiro–Wilk test, while the homogeneity of residual variances across treatments was examined by Levene’s test. Variables that met both the residual normality and variance homogeneity assumptions were subjected to one-way ANOVA, with Duncan’s multiple range test subsequently applied for multiple comparisons among treatments. Differences were regarded as significant when *p* < 0.05, whereas 0.05 ≤ *p* < 0.10 was interpreted as a tendency. In the tables, distinct lowercase superscript letters denote significant differences among treatments based on Duncan’s multiple range test.

When variables did not fully satisfy the assumptions of normality or homogeneity of variance, appropriate data transformation was considered before parametric analysis. If the assumptions were still not met after transformation, the Kruskal–Wallis test followed by Dunn’s post hoc test was used for group comparisons. Potential outliers were examined based on studentized residuals and were removed only when a clear technical, analytical, or recording error could be identified; otherwise, all observations were retained in the final analysis.

For the 16S rRNA gene sequencing data, alpha-diversity indices were contrasted among treatments by means of one-way ANOVA or the Kruskal–Wallis test, depending on how the data were distributed. Beta-diversity patterns were displayed through PCoA built on Bray–Curtis distance and tested for among-treatment differences using PERMANOVA (999 permutations) with pairwise BH-FDR correction; multivariate dispersion was checked by PERMDISP. Discriminative microbial taxa among treatments were identified using LEfSe, with an LDA score > 2.0 used as the threshold for microbial markers.

For untargeted metabolomic data, peak intensity tables were subjected to quality control filtering, missing-value processing, normalization, log transformation, and scaling before downstream analysis. Differential metabolite features were screened using the criteria of VIP > 1 from the OPLS-DA model and *p* < 0.05 from univariate analysis. Positive- and negative-ion modes were merged for downstream analyses, and duplicate metabolite annotations were retained according to the higher VIP value. KEGG enrichment analysis was performed using annotated differential metabolite features with available KEGG identifiers. Unless confirmed by authentic standards, metabolite annotations were treated as putative identifications; therefore, metabolite-level and pathway-level results were interpreted as exploratory.

For integrated microbiome–metabolome association analysis, Spearman’s rank correlation coefficients were calculated between differential genera and differential metabolites. Correlations with |r| >= 0.60 and *p* < 0.05 were retained for network visualization. Because of the limited sample size, the microbiome–metabolome association networks were interpreted as exploratory and hypothesis-generating rather than as evidence of direct causal relationships.

## 3. Results

### 3.1. Plasma Inflammatory Biomarkers

Comparative analysis of inflammatory markers in sheep is summarized in Table 3, which details the levels of lipopolysaccharide (LPS), tumor necrosis factor-alpha (TNF-α), and interleukin-1 beta (IL-1β) across the control, aged maize with *Laminaria japonica* polysaccharide (AML), and aged maize (AM) groups. Significant differences among treatments are highlighted by distinct superscript letters (*p* < 0.05).

Plasma inflammatory biomarkers differed significantly among treatments (Table 3). Compared with the control group (CK), the aged-maize group (AM) exhibited a marked increase in circulating endotoxin burden, as reflected by higher plasma LPS concentrations (0.428 vs. 0.379 EU/mL; *p* < 0.001). Strikingly, LJP supplementation in the aged-maize diet (AML) significantly reduced LPS to 0.336 EU/mL (*p* < 0.001), falling below both AM and CK. A similar pattern was observed for TNF-α: AM showed the highest concentration (45.77 pg/mL), CK was intermediate (42.07 pg/mL), and AML displayed the lowest level (35.73 pg/mL; *p* < 0.001). Plasma IL-1β also differed among groups (*p* = 0.0184), with AML significantly lower than AM (22.94 vs. 27.59 pg/mL), whereas CK remained intermediate (24.61 pg/mL) and was not distinguishable from either group based on Duncan’s multiple range test (superscript “ab”). Overall, plasma LPS, TNF-α, and IL-1β differed among treatments, with AM showing the highest values and AML the lowest.

### 3.2. Intestinal Antioxidant Indices

Antioxidant parameters in the ileum and colon of Hu sheep, influenced by normal control CK), aged maize (AM), and aged maize supplemented with LJP (AML) diets, are outlined in Table 4.

Several intestinal antioxidant and oxidative-stress indices differed among dietary treatments in both the ileum and colon (Table 4). In the ileum, aged maize (AM) increased oxidative stress-related indices, as evidenced by a pronounced elevation in ROS relative to the control (CK) (248.79 vs. 166.23 fluorescence intensity/mg; *p* < 0.001), accompanied by higher SOD activity (26.89 vs. 20.48 U/mL; *p* < 0.001) and increased MDA (1.87 vs. 1.63 nmol/L; *p* = 0.006). Notably, supplementation with *Laminaria japonica* polysaccharide (AML) showed higher GSH-Px and T-AOC and lower ROS and MDA than AM: AML elevated GSH-Px and T-AOC in a stepwise manner (GSH-Px: 48.41 > 45.15 > 40.31 μmol/L; T-AOC: 3.10 > 2.60 > 2.30 U/mL for AML > AM > CK; both *p* < 0.001), while reducing ROS compared with AM (188.61 vs. 248.79; *p* < 0.001) and lowering MDA to below CK (1.48 vs. 1.63 nmol/L; *p* = 0.006). A similar pattern was observed in the colon. Aged maize significantly increased ROS (270.69 vs. 224.09; *p* < 0.001) and induced higher SOD (23.27 vs. 17.97 U/mL; *p* < 0.001), whereas AML further enhanced antioxidant capacity—reflected by elevated T-AOC (2.26 > 1.89 > 1.66 U/mL; *p* < 0.001) and higher GSH-Px relative to CK (*p* = 0.003)—and concomitantly reduced ROS compared with AM (247.78 vs. 270.69; *p* < 0.001). Moreover, colonic MDA was lower in AML than in AM and CK (1.64 vs. 1.81–1.98 nmol/L; *p* = 0.049).

### 3.3. Plasma Biochemical Indices

The plasma biochemical metabolites of Hu sheep across the CK, AM, and AML treatments are listed in Table 5.

Plasma biochemical indices are shown in Table 5. TC, TG, HDL, LDL, ALT, TBIL, and ALP did not differ significantly among treatments (*p* > 0.05), whereas AST and DBIL differed among groups. Concentrations of total cholesterol (TC), triglycerides (TG), HDL, and LDL did not differ among groups (*p* ≥ 0.334), indicating that the dietary treatments did not markedly perturb systemic lipid metabolism under the present conditions. In contrast, liver-associated enzymes showed evidence of mild functional challenge: plasma AST was significantly higher in the aged-maize group (AM) than in the control (CK) (144.72 vs. 111.68 U/L; *p* = 0.033), while the polysaccharide-supplemented group (AML) displayed an intermediate value (127.78 U/L) that was not distinguishable from either AM or CK based on Duncan’s multiple range test (superscript “ab”). Similarly, direct bilirubin (DBIL) was significantly increased in both AM and AML compared with CK (1.92 and 1.81 vs. 1.45 μmol/L; *p* = 0.008), whereas total bilirubin (TBIL) and ALP did not differ significantly among treatments (*p* > 0.05). ALT did not differ among groups (*p* = 0.766).

### 3.4. Hepatic Antioxidant Indices

Hepatic antioxidant indices for Hu sheep across the CK, AM, and AML groups are presented in Table 6.

Hepatic antioxidant indices differed selectively among treatments (Table 6). Total antioxidant capacity (T-AOC) was significantly higher in the polysaccharide-supplemented group (AML) than in both CK and AM (2.33 vs. 1.95–1.98 U/mL; *p* = 0.009). Catalase (CAT) activity also differed among groups (*p* = 0.013), with AML showing a higher value than CK (3.91 vs. 2.75 U/mL) and AM exhibiting an intermediate level (3.22 U/mL; superscript “ab”). In contrast, SOD activity, GSH-Px activity, and MDA concentration were not significantly affected by treatment (*p* = 0.109, 0.622, and 0.352, respectively), although MDA was numerically lower in AML than in CK and AM.

### 3.5. Cecal Metabolomic Profiling

**Figure 1:**
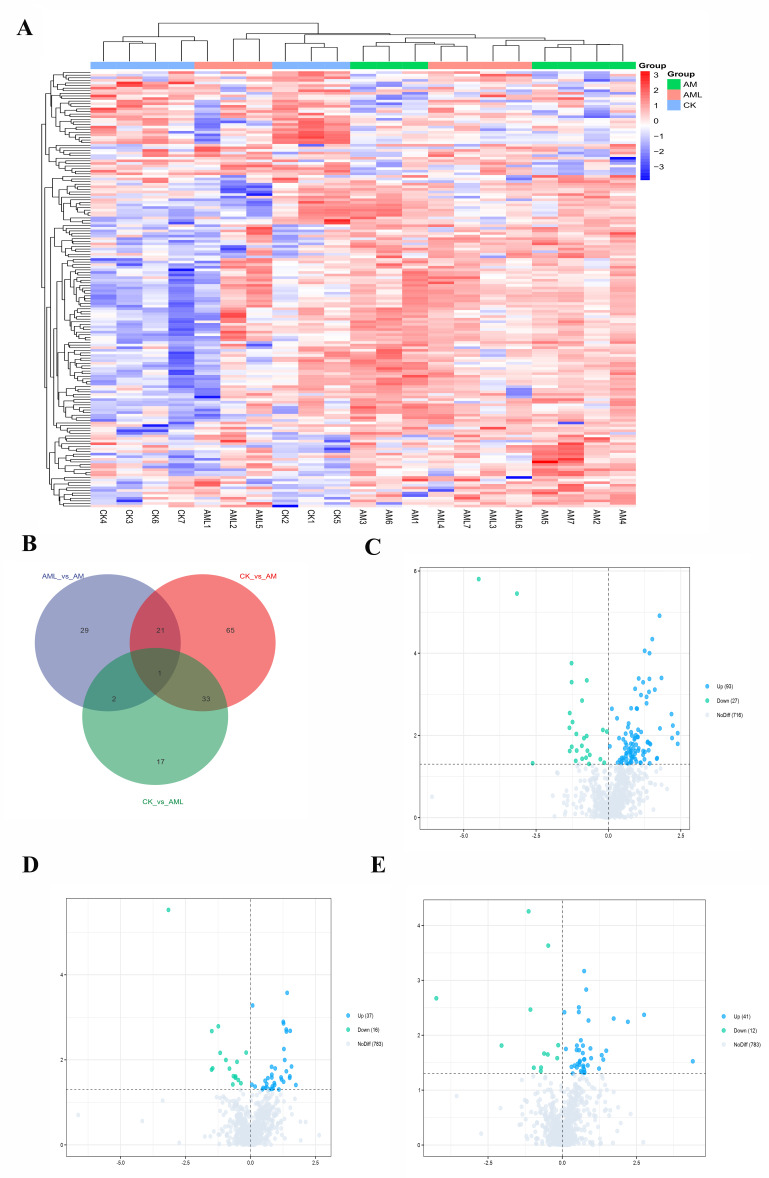
Cecal metabolomic remodeling among groups and the mitigating effect of seaweed polysaccharide. (**A**) Unsupervised hierarchical clustering heatmap of differential metabolites. (**B**) Venn diagram showing overlap of differential metabolites among pairwise comparisons. (**C**–**E**) Volcano plots for CK vs. AM, CK vs. AML, and AML vs. AM, respectively.

Untargeted metabolomic profiling identified differential metabolite features in cecal contents among the dietary groups (Figure 1). The hierarchical clustering heatmap (Figure 1A) displayed the abundance patterns of differential metabolite features across CK, AM, and AML samples. The Venn diagram (Figure 1B) showed 65, 17, and 29 comparison-specific differential metabolite features in CK vs. AM, CK vs. AML, and AML vs. AM, respectively, with one feature shared by all three pairwise comparisons. Volcano plots (Figure 1C–E) showed that CK vs. AM contained 120 differential metabolite features, including 93 upregulated and 27 downregulated features; CK vs. AML contained 53 features, including 37 upregulated and 16 downregulated features; and AML vs. AM contained 53 features, including 41 upregulated and 12 downregulated features.

### 3.6. Differential Cecal Metabolite Features and Clustering Patterns

To further describe the differential metabolite features identified across treatments, representative cecal metabolite features were examined using hierarchical clustering. These features displayed distinct abundance patterns among CK, AM, and AML samples, suggesting that aged-maize feeding and LJP supplementation were accompanied by coordinated changes in selected cecal metabolite profiles. The clustered features included MP10239, MP10352, MP10399, MP10473, MP10707, MP10795, and MP10814, which were putatively annotated as 4-Methylpropranolol, 1-(4-Isopropoxyphenyl)-3-(1-piperidinyl)-1-propanone, 6-[3]-ladderane-hexanoic acid, 5-hydroxyindole thiazolidine carboxylate, 2-(3-{[3-(Tetrahydro-2H-pyran-4-ylamino)-3-oxetanyl]methyl}-1,2-oxazol-5-yl)ethanol, 3,3-Dibromo-2-n-butylacrylic acid, and 5,7-Methoxyflavanone, respectively. Because these annotations were derived from untargeted metabolomics, these compounds were interpreted as putatively annotated metabolite features rather than confirmed metabolites.

### 3.7. KEGG Enrichment Analysis of Differential Cecal Metabolite Features

**Figure 2:**
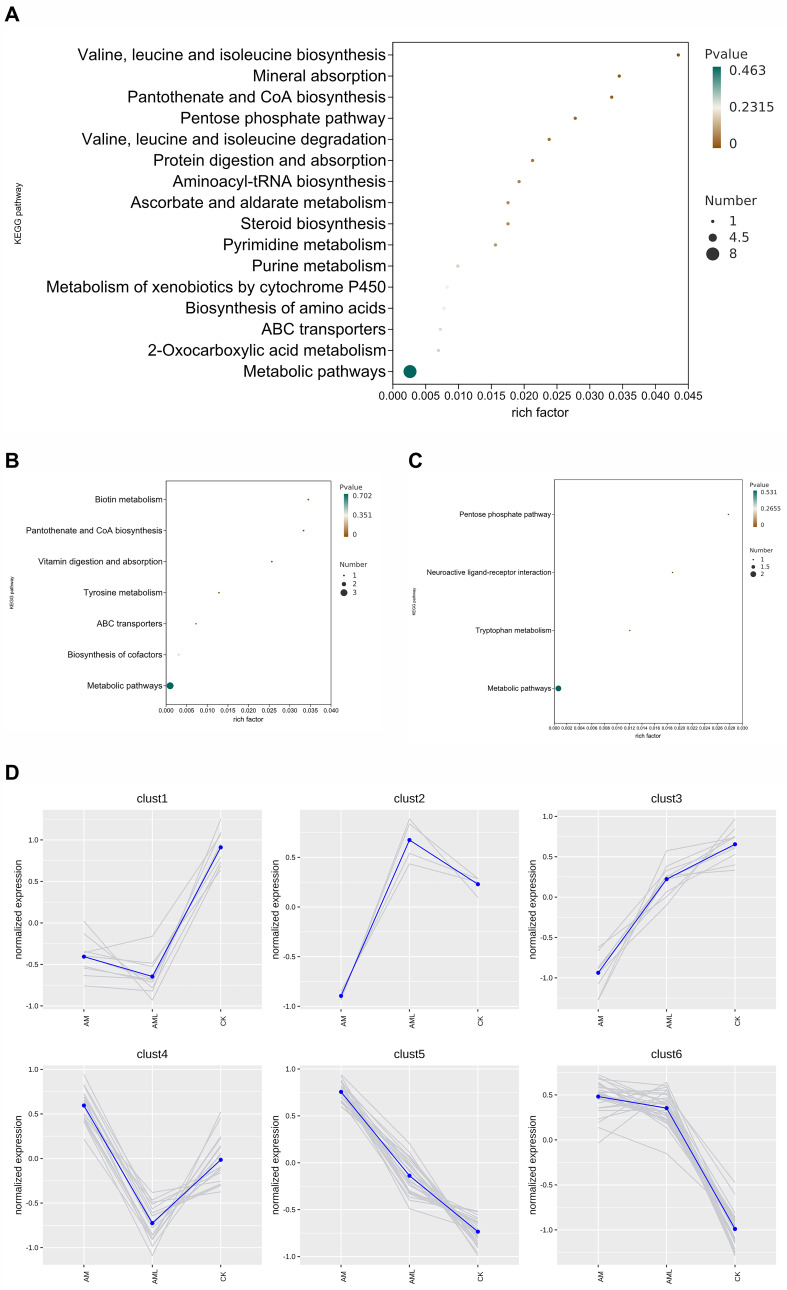
KEGG pathway enrichment and abundance trends of differential cecal metabolites. (**A**–**C**) KEGG pathway enrichment analysis of differential metabolites for CK vs. AM, CK vs. AML, and AML vs. AM. Dot color represents the *p*-value, and dot size represents the number of differential metabolites mapped to each pathway. (**D**) Trend analysis of the differential metabolites across the three dietary groups. Differential metabolites were grouped into six clusters (clust1–clust6) by hierarchical clustering (Euclidean distance, complete linkage) of their group-mean profiles with Z-score standardization. Grey lines denote individual metabolites and the blue line indicates the cluster mean. The x-axis shows the dietary groups (AM, AML, CK) and the y-axis represents the normalized (Z-score) metabolite abundance.

KEGG enrichment analysis was performed to further characterize pathway-level changes associated with aged-maize feeding and LJP supplementation (Figure 2A–C). In the CK vs. AM comparison, differential metabolite features were mainly assigned to purine metabolism, metabolism of xenobiotics by cytochrome P450, biosynthesis of amino acids, ABC transporters, 2-oxocarboxylic acid metabolism, and general metabolic pathways. Representative annotated features included hypoxanthine, L-isoleucine, 2-deoxy-D-ribose, glucuronolactone, cytosine, and several xenobiotic- or lipid-related features. These results suggest that aged-maize feeding was associated with broad shifts in nucleotide metabolism, amino acid-related metabolism, carbohydrate-related metabolism, and xenobiotic-associated metabolic processes.

In the AM vs. AML comparison, differential metabolite features were enriched in the pentose phosphate pathway, tryptophan metabolism, neuroactive ligand–receptor interaction, and general metabolic pathways. The pentose phosphate pathway involved 2-deoxy-D-ribose, whereas tryptophan metabolism and neuroactive ligand–receptor interaction involved tryptamine. Because several enriched pathways showed borderline or non-significant FDR-adjusted values, these results were interpreted as candidate metabolic signatures rather than definitive pathway-level alterations.

In the CK vs. AML comparison, enriched pathways included biotin metabolism, pantothenate and CoA biosynthesis, vitamin digestion and absorption, tyrosine metabolism, ABC transporters, biosynthesis of cofactors, and general metabolic pathways. Representative annotated features included biotin, D-panthenol, and DL-metanephrine. This pattern suggests that the AML group did not simply return to the CK metabolic profile, but displayed a distinct cecal metabolic configuration involving vitamin/cofactor-related and amino acid-related pathways. Together with the trend analysis of differential metabolite features across the three dietary groups (Figure 2D), these pathway-level results support treatment-associated cecal metabolomic remodeling, while remaining exploratory because the analysis was based on untargeted metabolite annotations.

### 3.8. Cecal Microbiota Composition and Predicted Functions

**Figure 3:**
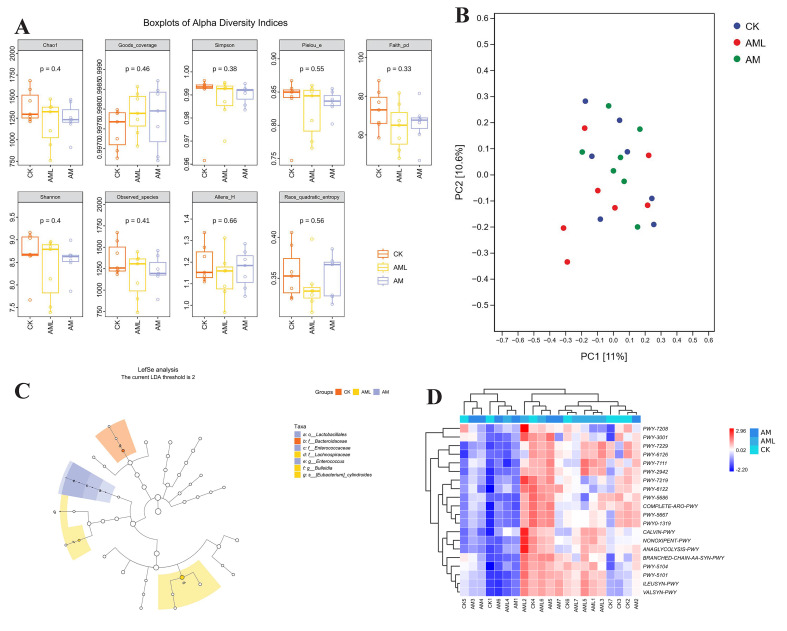
Seaweed polysaccharide modulates cecal microbiota structure and predicted functions in sheep fed an aged-maize diet. (**A**) Alpha-diversity indices. (**B**) PCoA based on Bray–Curtis dissimilarity; among-group difference was non-significant (PERMANOVA *p* = 0.37). (**C**) LEfSe cladogram showing discriminative taxa (LDA > 2). (**D**) Heatmap of differential predicted microbial functional pathways.

Cecal 16S rRNA profiling indicated that dietary treatments were associated with changes in specific taxa and predicted functions rather than with overall community diversity or structure (Figure 3). Alpha-diversity indices (Chao1, Shannon, Simpson, Pielou’s evenness, Faith’s phylogenetic diversity, and related metrics) did not differ significantly among CK, AM, and AML (all *p* > 0.33; Figure 3A), indicating comparable richness and evenness. Overall community structure was also not significantly different among groups (PERMANOVA on Bray–Curtis distances: pseudo-F = 1.02, R^2^ = 0.10, *p* = 0.37; all pairwise *p* > 0.05 after BH-FDR correction; PERMDISP *p* = 0.51). Thus, although PCoA suggested a visual gradient along the first two coordinates (PC1 = 11.0%, PC2 = 10.6%; Figure 3B), this separation was not statistically supported. Treatment-associated differences were instead evident at finer resolution: LEfSe identified discriminative taxa among groups (LDA > 2; Figure 3C), and predicted functional profiling revealed group-associated pathway-level patterns (Figure 3D).

### 3.9. Discriminative Cecal Microbial Markers Associated with Dietary Treatments

LEfSe analysis was used to identify discriminative cecal microbial markers among the three dietary treatments. The CK group was characterized by enrichment of Bacteroidaceae, whereas the AM group was mainly characterized by taxa within Lactobacillales, Enterococcaceae, and Enterococcus. In contrast, the AML group was characterized by Lachnospiraceae, Bulleidia, and Eubacterium_cylindroides. Because LEfSe was used as a biomarker discovery method, these taxa were interpreted as treatment-associated microbial markers rather than definitive causal drivers.

### 3.10. Integrated Microbiome–Metabolome Association Patterns

**Figure 4:**
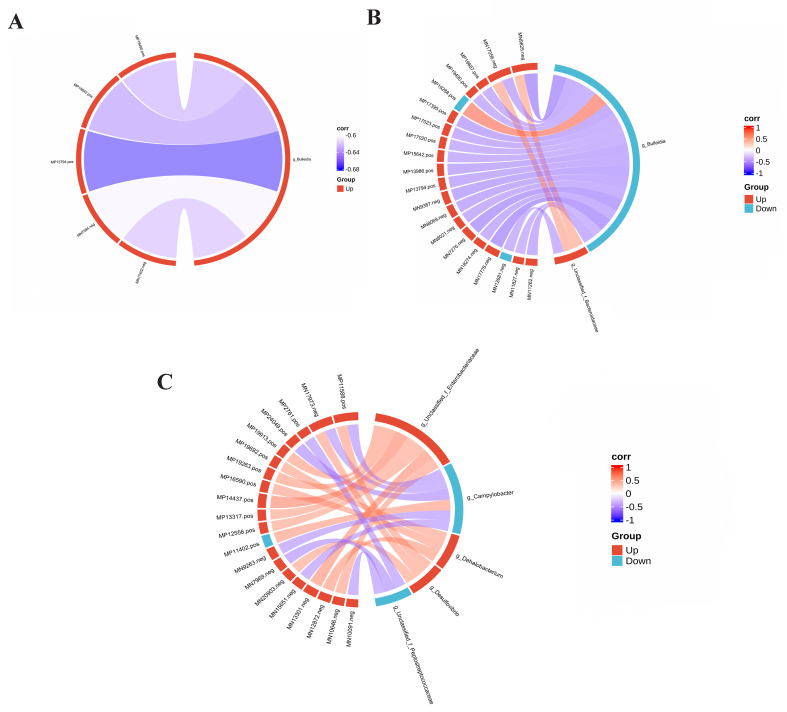
Integrated microbiome–metabolome associations in cecal contents reveal diet- and polysaccharide-dependent microbial–metabolic coupling.(**A**–**C**) Circos chord diagrams showing significant correlations (Spearman’s correlation coefficients) between differential genera (16S rRNA) and differential metabolites (untargeted metabolomics) in CK vs. AML (**A**), AM vs. AML (**B**), and CK vs. AM (**C**). Ribbon color denotes correlation direction/strength, and the outer track indicates metabolites classified as up- or down-regulated in the corresponding comparison.

Integrated microbiome–metabolome correlation analysis was performed to assess associations between differential genera and differential metabolite features (Figure 4A–C). Circos chord diagrams summarized significant Spearman correlations between differential genera and differential metabolite features, using |r| ≥ 0.60 and *p* < 0.05 as filtering criteria and retaining the top 20 taxa and top 20 metabolites for visualization. In the CK vs. AML network, Bulleidia was associated with a limited set of metabolite features. In the AM vs. AML network, Bulleidia and an unclassified Bacteroidaceae lineage were connected with multiple metabolite features through both positive and negative correlations. In the CK vs. AM network, Campylobacter, Desulfovibrio, and additional genera were associated with multiple differential metabolite features. Because the network was based on Spearman correlation analysis, these associations should be interpreted as exploratory treatment-associated patterns rather than evidence of direct causal interactions.

## 4. Discussion

A principal contribution of the present study is the evidence that aged-maize feeding was associated not only with changes in isolated biochemical indices, but also with coordinated alterations across gut- and liver-related endpoints in Hu sheep. The AM diet increased circulating LPS, TNF-α, and IL-1β, elevated intestinal ROS and lipid peroxidation, altered cecal microbiota and metabolite profiles, and was accompanied by selective hepatobiliary changes, whereas LJP shifted several of these indices in the opposite direction. Viewed integratively, these findings support a working model in which aged maize may disturb hindgut redox and ecological homeostasis, thereby contributing to microbial-product translocation, systemic inflammatory pressure, and a secondary hepatic antioxidant/biliary response. This interpretation is consistent with contemporary concepts of gut–liver crosstalk, in which intestinal barrier integrity, microbial metabolites, LPS signaling, and hepatic stress responses form a tightly coupled pathophysiological network [14,34,35,36].

### 4.1. Aged-Maize Feeding Was Associated with Increased Endotoxin Exposure and Systemic Inflammation

The increase in plasma LPS in AM sheep is consistent with enhanced endotoxin exposure. In gut–liver-axis biology, LPS is commonly considered a marker of impaired microbial containment, increased mucosal permeability, or both, and it can amplify TLR4/NF-κB-driven cytokine release and inflammatory signaling beyond the intestine. The concurrent increases in TNF-α and IL-1β therefore suggest that aged maize was associated with gut-derived inflammatory pressure rather than a purely local intestinal response. The simultaneous rise in circulating endotoxin and cytokines indicates that the response was not confined to the mucosa but extended systemically along the gut–liver axis. LJP reduced all three indices, with LPS falling below the control level. This profile suggests that LJP may have attenuated inflammatory signaling at least in part by limiting the generation and/or translocation of pro-inflammatory microbial products.

Although the proximate injurious factor in aged maize was not chemically resolved in the present study, the cecum of sheep is known to be highly responsive to diet-driven perturbation, and compromised feed quality can provoke oxidative, inflammatory, and microbiota-related disturbances in ruminants [9,37]. Accordingly, we avoid attributing the AM phenotype to a single chemical trigger—whether lipid oxidation products, altered nutrient availability, microbial spoilage, or associated toxicants. What the present data show is that aged-maize feeding was associated with a coordinated shift toward endotoxemia, oxidative stress, and hepatobiliary strain, a pattern compatible with disrupted intestinal homeostasis.

### 4.2. The Intestine Appeared to Be a Major Responsive Site, and LJP Improved Redox-Related Antioxidant Capacity

The intestine appeared to be a major responsive tissue. Both the ileum and colon of sheep fed the aged-maize diet (AM) exhibited increased ROS, and the ileum additionally showed higher MDA compared with the control diet (CK), suggesting intestinal redox disequilibrium and lipid peroxidation under aged-maize feeding conditions. In the AM diet, the concomitant increase in SOD activity is best interpreted as an adaptive response to excessive superoxide generation rather than evidence of improved antioxidant status [38,39,40]. Importantly, antioxidant enzyme activities (SOD, GSH-Px) and oxidative damage markers (ROS, MDA) should not be interpreted interchangeably: the former reflect the capacity of—or a compensatory response by—the antioxidant defense system, whereas the latter directly index oxidative injury. In AM sheep, the parallel elevation of ROS and MDA together with increased SOD activity is therefore most consistent with oxidative stress accompanied by compensatory enzyme induction, rather than with improved antioxidant status. We cannot exclude the alternative interpretation that the elevated enzyme activities reflect a successful adaptive response that limited net damage; however, the concurrent accumulation of ROS and MDA indicates that detoxification capacity was, at least in part, exceeded. Once oxidant production exceeds detoxification capacity, compensatory enzyme induction can coexist with ongoing lipid peroxidation and may contribute to barrier vulnerability. Recent work has repeatedly linked oxidative injury to loss of tight-junction integrity, enhanced paracellular leakiness, and activation of redox-sensitive inflammatory programs, particularly those coordinated through the Nrf2 and NF-κB axes [41,42,43].

Against this backdrop, the LJP response is biologically coherent. LJP lowered intestinal ROS and MDA while augmenting GSH-Px and T-AOC, suggesting both a reduction in oxidative burden and reinforcement of endogenous buffering capacity. The magnitude of these shifts supports their biological relevance: aged maize raised ileal ROS by ≈50% above control, accompanied by smaller increases in ileal MDA (≈15%) and plasma LPS (≈13%), while LJP reversed these changes to a comparable degree (relative to AM, ileal ROS and MDA fell by ≈24% and ≈21%, and plasma LPS, TNF-α, and IL-1β by ≈21%, ≈22%, and ≈17%). Given that even modest but sustained elevations in circulating LPS can shape systemic inflammatory tone, reductions of this size are plausibly meaningful rather than merely statistically detectable. This pattern indicates that LJP improved the balance between oxidant generation and antioxidant defense rather than merely quenching terminal oxidation products. Although the present study did not quantify tight-junction proteins or permeability tracers, the concordance between improved intestinal redox indices and reduced circulating LPS is consistent with a putative barrier-related contribution. In other words, improved redox homeostasis may be one upstream component by which LJP helps limit endotoxin leakage and systemic inflammatory escalation [38,41,44].

### 4.3. LJP May Function as a Gut-Active Marine Polysaccharide Associated with Microbial Fermentation and Barrier-Supportive Metabolism

An important aspect of the present work is that LJP is not only an antioxidant additive in the narrow chemical sense; it may also behave as a gut-active marine polysaccharide with ecological and metabolic effects. Seaweed polysaccharides are only partially digested by the host and can therefore reach distal intestinal compartments, where they may serve as substrates for specialized microbial guilds and reshape cross-feeding networks [45,46]. In ruminants, this distal delivery presupposes at least partial escape from ruminal fermentation. Ruminal microbes readily ferment non-sulfated storage glucans (laminarin) and alginate, whereas the sulfated fraction (fucoidan) is hydrolyzed only to a limited extent even by seaweed-adapted rumen microbiota [47]. Given that the LJP used here was highly sulfated (sulfate group content 28.9%), a substantial sulfated fraction may partly bypass ruminal fermentation and reach the hindgut, where it remains available to cecal microbiota; the precise ruminal degradation of LJP was not measured in the present study and remains to be quantified [47]. Recent studies with Laminaria/Saccharina-derived polysaccharides show that these compounds can enrich polysaccharide-utilizing or SCFA-associated taxa, elevate fecal or cecal acetate, propionate, and butyrate, and improve host metabolic and inflammatory phenotypes [48,49,50]. The 2025 digestion-imaging study of LJP further supports this interpretation by showing that LJP persists through the gastrointestinal tract and promotes SCFA accumulation, supporting its characterization as a candidate prebiotic-like substrate rather than only a transient luminal compound [20].

This framework also helps explain a subtle feature of our microbiota data: alpha diversity remained stable, and overall community structure was not significantly altered, whereas specific taxa and metabolites showed treatment-associated changes. In dietary ecology, this pattern can indicate selective community reorganization rather than wholesale richness loss. That is, the challenge and the intervention may have altered the identity and functional relations of key taxa, even if total richness/evenness were preserved. Comparable cecal restructuring has been described in sheep subjected to diet-driven hindgut disturbance [9], and LJP-based multi-omics studies likewise show that *Laminaria polysaccharides* remodel the microbiota–metabolite interface without necessarily causing dramatic changes in global diversity metrics [16,18,24]. Within this conceptual frame, the AML group may represent a compositionally re-patterned ecosystem with altered predicted functional potential, rather than simply a partially corrected AM group.

### 4.4. Metabolite Remodeling May Help Explain the Incomplete Normalization of DBIL

Because the present metabolomics was untargeted, caution is warranted in naming individual mediators. Nevertheless, the current literature provides an interpretive scaffold for the pathway-level shifts observed here. First, SCFAs are plausible candidate mediators of the LJP response: they support epithelial energy metabolism, reinforce tight-junction assembly, and restrain inflammatory signaling, thereby potentially reducing LPS escape into circulation [15,16,17,18,19,20,24]. Second, microbial tryptophan catabolism deserves consideration. Tryptophan-derived indoles activate AhR-dependent programs that support epithelial differentiation, antimicrobial tone, and mucosal repair; conversely, loss of indole signaling can exacerbate liver injury through weakened barrier control [12,51,52,53,54]. Third, bile acids represent another plausible pathway. Gut bacteria regulate bile-acid deconjugation and secondary transformation, while bile acids in turn shape microbial ecology and signal through FXR/TGR5 to influence barrier integrity, inflammation, and hepatic metabolism.

This research is particularly relevant to the partial dissociation observed in our study: LJP lowered endotoxemia and substantially improved antioxidant indices, yet DBIL remained elevated. Rather than undermining the protective interpretation, this finding suggests that hepatobiliary recovery may follow kinetics distinct from those of inflammatory tone. We hypothesize that sustained high DBIL in AML may be associated with intestinal bile acid remodeling; targeted bile acid quantification is needed to verify this inference in follow-up trials. Similar polysaccharide- and microbiota-based interventions have shown that improvement in liver injury can coexist with ongoing adaptation of the bile-acid/FXR/FGF15 axis [26,27,28,29]. Thus, the persistent DBIL response in AML may reflect an unresolved or still-remodeling biliary component rather than failure of the intervention per se.

### 4.5. Hepatic Changes Suggest Secondary Liver Involvement

The hepatic phenotype in our study is best described as secondary but physiologically relevant. The increase in AST, in the absence of a parallel ALT rise, argues against frank hepatocellular necrosis yet remains compatible with mild hepatic stress under enhanced gut-derived inflammatory load. Within the gut–liver axis, even modest increases in portal LPS and inflammatory mediators can increase hepatic oxidative demand, engage Kupffer-cell signaling, and sensitize biliary and metabolic pathways before overt parenchymal injury becomes apparent [1,2,3,26,27,28,29]. In that sense, the liver data complement rather than duplicate the intestinal data: they suggest that the intestinal response may have systemic relevance, but not that advanced hepatic failure occurred within the experimental period.

The improvement of hepatic T-AOC and CAT in AML is therefore relevant to the overall response pattern. Catalase is a major peroxide-detoxifying enzyme, and its induction suggests enhanced capacity to intercept oxidative propagation within the liver. This response was of appreciable size (hepatic T-AOC and CAT ≈20% and ≈42% higher in AML than in control), consistent with a genuine increase in peroxide-detoxifying reserve rather than a marginal fluctuation. This response could reflect two non-exclusive possibilities: attenuation of upstream gut-derived pro-oxidant and inflammatory input, and enhancement of hepatic antioxidant resilience, both of which have been reported for *Laminaria* or other marine polysaccharides in metabolic liver injury models [55]. Importantly, the lack of significant changes in hepatic MDA, SOD, or GSH-Px does not exclude a beneficial response; rather, it may indicate that the liver was a secondary target and that the key effect of LJP was to increase reserve capacity before broad-spectrum enzymatic exhaustion or lipid peroxidation occurred. Conversely, some statistically significant differences were of limited absolute magnitude and warrant cautious interpretation: direct bilirubin differed among groups by only ≈0.5 μmol/L and remained within a low physiological range, and the ≈30% rise in plasma AST in AM occurred without a parallel ALT increase and from a moderate baseline. These hepatobiliary changes are therefore best regarded as mild and of uncertain practical importance, which tempers the strength of any mechanistic claim about liver involvement.

### 4.6. Integrated Microbiome–Metabolome Networks Suggest Treatment-Associated Microbial–Metabolic Remodeling

The integrated microbiome–metabolome analysis provides exploratory association-level insight that single-endpoint biochemistry alone could not provide. These association patterns were examined independently of overall community-level testing and are descriptive in nature. In AM sheep, the cecal network displayed more extensive microbial–metabolic coupling, whereas AML generated a distinct association architecture. We interpret this not as a simple gain or loss of interactions, but as potential ecological rewiring: aged maize may have favored a stress-associated network compatible with inflammatory and oxidative outputs, while LJP may have redirected that network toward a more buffered and metabolically constructive state. Such an interpretation is consistent with recent multi-omics studies in which marine polysaccharides reshape cecal or fecal metabolite signatures in parallel with selective microbial restructuring, yielding host-related benefits that are more clearly described when taxa and metabolites are analyzed as integrated modules rather than separate layers [16,30].

The taxa highlighted in our network analysis are also biologically plausible. Desulfovibrio overgrowth has been reported to be linked to increased tight-junction permeability [56], while Campylobacter is a well-established barrier-disruptive enteropathogen whose injurious effects can be mitigated by barrier-supportive nutritional or synbiotic strategies [57,58]. We would nonetheless avoid causal language here: the present data support association, not direct causation. The safest high-level interpretation is that aged maize favored a microbial context compatible with barrier stress and pro-inflammatory metabolite flux, whereas LJP remodeled that context into one potentially less conducive to endotoxin spillover and oxidative propagation.

The KEGG enrichment results provide additional, although exploratory, support for treatment-associated cecal metabolic remodeling. In particular, the AM vs. AML comparison highlighted the pentose phosphate pathway and tryptophan-related metabolism, represented by 2-deoxy-D-ribose and tryptamine, respectively. These pathways are biologically plausible in the context of intestinal redox balance and host–microbe immunometabolic interactions. However, because the present metabolomic analysis was untargeted and pathway enrichment was based on a limited number of annotated features, these results should be regarded as candidate metabolic directions rather than confirmed mechanisms. Future targeted validation of tryptophan metabolites, short-chain fatty acids, bile acids, and redox-related metabolites is needed to clarify the metabolic contribution of LJP under aged-maize feeding conditions.

## 5. Conclusions

Under the present conditions, replacing normal maize with aged maize was associated with elevated circulating LPS and pro-inflammatory cytokines, intestinal oxidative stress, mild liver-related biochemical changes, and cecal microbiome–metabolome remodeling in Hu sheep. Dietary *Laminaria japonica* polysaccharide partially counteracted these responses, lowering endotoxin and inflammatory markers, improving intestinal and hepatic antioxidant capacity, and reshaping cecal microbe–metabolite associations.

These conclusions remain associative. The interpretation of coordinated intestinal and hepatic changes is inferential, as no direct measures of intestinal barrier function, intestinal histology, or liver histopathology were obtained; growth performance and ruminal fermentation were not assessed and the microbiome–metabolome associations—based on correlation networks, putative metabolite annotations, and predictive functional inference—are hypothesis-generating rather than causal. With a modest sample size (*n* = 7) and a single LJP dose (0.5% DM), dose–response inference is not possible. Future work combining direct barrier and histological assessment, targeted metabolite quantification, growth-performance data, and multiple doses is needed to confirm the mechanisms suggested here.

## Figures and Tables

**Table 1:** Safety-related contaminant and hygienic indicators of the aged maize compared with the maximum limits specified in GB 13078-2017.

Item	Measured Value	Maximum Limit (GB 13078-2017)
Mycotoxins		
Aflatoxin B_1_	3.6 μg/kg	≤30 μg/kg
Zearalenone, ZEN	0.12 mg/kg	≤0.5 mg/kg
Deoxynivalenol, DON	0.62 mg/kg	≤5 mg/kg
Ochratoxin A	<2.0 μg/kg	≤100 μg/kg
T-2 toxin	0.15 mg/kg	≤0.5 mg/kg
Fumonisins	0.85 mg/kg	≤60 mg/kg
Heavy metals		
As	0.45 mg/kg	≤2.0 mg/kg
Pb	1.20 mg/kg	≤10 mg/kg
Hg	0.015 mg/kg	≤0.1 mg/kg
Cd	0.08 mg/kg	≤1 mg/kg
Other inorganic contaminants		
F	8.5 mg/kg	≤150 mg/kg
Nitrite (as NaNO_2_)	3.2 mg/kg	≤15 mg/kg
Cyanide (as HCN)	<2.0 mg/kg	≤50 mg/kg
Organochlorine pollutants and pesticide residues		
Polychlorinated biphenyls (PCBs)	<5.0 µg/kg	≤10 µg/kg
Hexachlorocyclohexane (HCH)	<0.01 mg/kg	≤0.2 mg/kg
Dichlorodiphenyltrichloroethane (DDT)	<0.01 mg/kg	≤0.05 mg/kg
Hexachlorobenzene (HCB)	<0.005 mg/kg	≤0.01 mg/kg
Antinutritional factors		
Free gossypol	Not detected	≤20 mg/kg
Isothiocyanate	Not detected	≤100 mg/kg
Oxazolidinethione	Not detected	Rapeseed-specific; no maize limit
Microbiological indicators		
Total mold count	3.5 × 10^4^ CFU/g	≤4 × 10^4^ CFU/g
Salmonella	Not detected	Not permitted

**Table 2:** Composition and nutrient levels of the trial basal diets (DM basis).

Item	CK	AM
Ingredient composition (%DM)		
Chinese wildrye hay	30	30
Normal maize	32.76	0
Aged maize	0	32.76
Sprayed maize bran	14	14
Maize germ meal	3.5	3.5
DDGS ^1^	10.5	10.5
Soybean meal	5.6	5.6
Beet molasses	0.7	0.7
Limestone	1.05	1.05
Slow-released NH_4_Cl	0.42	0.42
NaCl	0.42	0.42
CaHPO_4_	0.42	0.42
NaHSO_4_	0.35	0.35
Compound premix	0.28	0.28
Total	100	100
Nutrient levels (%DM)		
DM	95.10	94.73
CP	16.22	16.78
EE	4.33	3.86
Ash	4.51	4.69
NDF	21.14	21.27
ADF	4.59	4.78

Note: ^1^ DDGS: dry distiller’s grains and its solubles. Compound premix per kilogram contains vitamin A 1500 IU, vitamin D 200 IU, vitamin E 15 IU, iron 75 mg, copper 50 mg, manganese 40 mg, zinc 50 mg, selenium 0.5 mg, iodine 1.0 mg, cobalt 0.5 mg. Nutritional level was the detection value.

**Table 3:** Analysis of differences in the test results of inflammatory factors in sheep.

Project	Treatment			Standard Error	*p* Value
Item	CK	AM	AML	SEM	
plasma					
LPS (EU/mL)	0.379 ^b^	0.428 ^a^	0.336 ^c^	0.0112	<0.001
TNFα (pg/mL)	42.07 ^b^	45.77 ^a^	35.73 ^c^	1.093	<0.001
IL-1β (pg/mL)	24.61 ^ab^	27.59 ^a^	22.94 ^b^	1.049	0.0184

Note: CK, control diet containing normal maize; AM, aged-maize diet; AML, aged-maize diet supplemented with 0.5% *Laminaria japonica* polysaccharide on a dry matter basis. SEM, standard error of the mean. Values are means, *n* = 7 per treatment. Within the same row, values with different superscript letters differ significantly at *p* < 0.05 according to Duncan’s multiple range test.

**Table 4:** Effects of replacing normal maize with aged maize and adding *Laminaria japonica* polysaccharide on intestinal antioxidant indexes of Hu sheep.

Project	Treatment			Standard Error	*p* Value
Item	CK	AM	AML	SEM	
ileum					
SOD (U/mL)	20.48 ^b^	26.89 ^a^	30.12 ^a^	1.235	<0.001
MDA (nmol/L)	1.63 ^b^	1.87 ^a^	1.48 ^b^	0.076	0.006
GSH-Px (μmol/L)	40.31 ^c^	45.15 ^b^	48.41 ^a^	0.968	<0.001
T-AOC (U/mL)	2.30 ^c^	2.60 ^b^	3.10 ^a^	0.086	<0.001
ROS (Fluorescence intensity/mg)	166.23 ^b^	248.79 ^a^	188.61 ^b^	12.862	<0.001
colon					
SOD (U/mL)	17.97 ^b^	23.27 ^a^	25.61 ^a^	0.983	<0.001
MDA (nmol/L)	1.81 ^a^	1.98 ^a^	1.64 ^b^	0.089	0.049
GSH-Px (μmol/L)	38.22 ^b^	41.78 ^a^	44.15 ^a^	1.049	0.003
T-AOC (U/mL)	1.66 ^c^	1.89 ^b^	2.26 ^a^	0.065	<0.001
ROS (Fluorescence intensity/mg)	224.09 ^c^	270.69 ^a^	247.78 ^b^	6.904	<0.001

Note: CK, control diet containing normal maize; AM, aged-maize diet; AML, aged-maize diet supplemented with 0.5% *Laminaria japonica* polysaccharide on a dry matter basis. SEM, standard error of the mean. SOD, superoxide dismutase; MDA, malondialdehyde; GSH-Px, glutathione peroxidase; T-AOC, total antioxidant capacity; ROS, reactive oxygen species. Values are means, *n* = 7 per treatment. Within the same row, values with different superscript letters differ significantly at *p* < 0.05 according to Duncan’s multiple range test.

**Table 5:** Effects of replacing normal maize with aged maize and adding *Laminaria japonica* polysaccharide on plasma metabolites of Hu sheep.

Project	Treatment			Standard Error	*p* Value
Item	CK	AM	AML	SEM	
biochemistry					
TC (mmol/L)	1.63	1.64	1.60	0.093	0.957
TG (mmol/L)	0.35	0.23	0.34	0.062	0.334
HDL-C (mmol/L)	0.69	0.68	0.68	0.049	0.986
LDL-C (mmol/L)	0.79	0.74	0.75	0.057	0.845
AST (U/L)	111.68 ^b^	144.72 ^a^	127.78 ^ab^	8.106	0.033
ALT (U/L)	24.38	22.78	23.12	1.589	0.766
TBIL (μmol/L)	3.40	3.03	3.16	0.124	0.141
DBIL (μmol/L)	1.45 ^b^	1.92 ^a^	1.81 ^a^	0.099	0.008
ALP (U/L)	273.51	211.68	227.46	21.841	0.151

Note: CK, control diet containing normal maize; AM, aged-maize diet; AML, aged-maize diet supplemented with 0.5% *Laminaria japonica* polysaccharide on a dry matter basis. SEM, standard error of the mean. TC, total cholesterol; TG, triglyceride; HDL-C, high-density lipoprotein cholesterol; LDL-C, low-density lipoprotein cholesterol; AST, aspartate aminotransferase; ALT, alanine aminotransferase; TBIL, total bilirubin; DBIL, direct bilirubin; ALP, alkaline phosphatase. Values are means, *n* = 7 per treatment. Within the same row, values with different superscript letters differ significantly at *p* < 0.05 according to Duncan’s multiple range test.

**Table 6:** Effects of replacing normal maize with aged maize and adding *Laminaria japonica* polysaccharide on liver antioxidant indexes of Hu sheep.

Project	Treatment			Standard Error	*p* Value
Item	CK	AM	AML	SEM	
SOD (U/mL)	11.91	14.18	12.34	0.769	0.109
MDA (nmol/mL)	0.31	0.34	0.26	0.037	0.352
GSH-Px (μmol/L)	12.34	12.16	13.59	1.134	0.622
T-AOC (U/mL)	1.95 ^b^	1.98 ^b^	2.33 ^a^	0.073	0.009
CAT (U/mL)	2.75 ^b^	3.22 ^ab^	3.91 ^a^	0.248	0.013

Note: CK, control diet containing normal maize; AM, aged-maize diet; AML, aged-maize diet supplemented with 0.5% *Laminaria japonica* polysaccharide on a dry matter basis. SEM, standard error of the mean. SOD, superoxide dismutase; MDA, malondialdehyde; GSH-Px, glutathione peroxidase; T-AOC, total antioxidant capacity; CAT, catalase. Values are means, *n* = 7 per treatment. Within the same row, values with different superscript letters differ significantly at *p* < 0.05 according to Duncan’s multiple range test.

## Data Availability

All data generated or analyzed during this study are included in this published article. Additional data related to this study are available from the corresponding author upon reasonable request.
